# Deliberate Attenuation of Chikungunya Virus by Adaptation to Heparan Sulfate-Dependent Infectivity: A Model for Rational Arboviral Vaccine Design

**DOI:** 10.1371/journal.pntd.0002719

**Published:** 2014-02-20

**Authors:** Christina L. Gardner, Jozef Hritz, Chengqun Sun, Dana L. Vanlandingham, Timothy Y. Song, Elodie Ghedin, Stephen Higgs, William B. Klimstra, Kate D. Ryman

**Affiliations:** 1 Center for Vaccine Research, University of Pittsburgh, Pittsburgh, Pennsylvania, United States of America; 2 Department of Microbiology & Molecular Genetics, University of Pittsburgh, Pittsburgh, Pennsylvania, United States of America; 3 CEITEC, Masaryk University, Brno, Czech Republic; 4 Department of Diagnostic Medicine & Pathobiology, Biosecurity Research Institute, Kansas State University, Manhattan, Kansas, United States of America; 5 Department of Computational and Systems Biology, University of Pittsburgh, Pittsburgh, Pennsylvania, United States of America; University of Texas Medical Branch, United States of America

## Abstract

Mosquito-borne chikungunya virus (CHIKV) is a positive-sense, single-stranded RNA virus from the genus *Alphavirus*, family Togaviridae, which causes fever, rash and severe persistent polyarthralgia in humans. Since there are currently no FDA licensed vaccines or antiviral therapies for CHIKV, the development of vaccine candidates is of critical importance. Historically, live-attenuated vaccines (LAVs) for protection against arthropod-borne viruses have been created by blind cell culture passage leading to attenuation of disease, while maintaining immunogenicity. Attenuation may occur *via* multiple mechanisms. However, all examined arbovirus LAVs have in common the acquisition of positively charged amino acid substitutions in cell-surface attachment proteins that render virus infection partially dependent upon heparan sulfate (HS), a ubiquitously expressed sulfated polysaccharide, and appear to attenuate by retarding dissemination of virus particles *in vivo*. We previously reported that, like other wild-type Old World alphaviruses, CHIKV strain, La Réunion, (CHIKV-LR), does not depend upon HS for infectivity. To deliberately identify CHIKV attachment protein mutations that could be combined with other attenuating processes in a LAV candidate, we passaged CHIKV-LR on evolutionarily divergent cell-types. A panel of single amino acid substitutions was identified in the E2 glycoprotein of passaged virus populations that were predicted to increase electrostatic potential. Each of these substitutions was made in the CHIKV-LR cDNA clone and comparisons of the mutant viruses revealed surface exposure of the mutated residue on the spike and sensitivity to competition with the HS analog, heparin, to be primary correlates of attenuation *in vivo*. Furthermore, we have identified a mutation at E2 position 79 as a promising candidate for inclusion in a CHIKV LAV.

## Author Summary

With the adaptation of chikungunya virus (CHIKV) to transmission by the *Aedes albopictus* mosquito, a pandemic has occurred resulting in four to six million human infections, and the virus continues to become endemic in new regions, most recently in the Caribbean. CHIKV can cause debilitating polyarthralgia, lasting for weeks to years, and there are currently no licensed vaccines or antiviral therapies available. While an investigational live-attenuated vaccine (LAV) exists, problems with reactogenicity have precluded its licensure. The purpose of the current study was to: i) devise an *in vitro* passage procedure that reliably generates a panel of CHIKV envelope glycoprotein mutations for screening as vaccine candidates; ii) determine the position of the mutations in the three-dimensional structure of the alphavirus spike complex and their effect on electrostatic potential; iii) determine the attenuation characteristics of each mutation in a murine model of CHIKV musculoskeletal disease; and iv) to identify *in vitro* assays examining the dependency of infection upon HS that correlate with attenuation and localization in the glycoprotein spike. This approach provides a paradigm for the rational design of future LAVs for CHIKV and other mosquito-borne viruses, by deliberately selecting and combining attenuating processes.

## Introduction

In the last few years, considerable attention has been focused upon mosquito-borne chikungunya virus (CHIKV); once a relatively obscure member of the *Alphavirus* genus in the *Togaviridae* family of enveloped, positive-sense RNA viruses [1]–[3]. In 2005, an East African clade CHIKV strain emerged on the Indian Ocean island of La Réunion that was maintained in a human-mosquito-human transmission cycle and caused a massive outbreak of CHIK fever [4]. Spread and/or re-emergence of CHIKV in Indian Ocean areas, Asia, southern Europe, and most recently in the Caribbean, in the following years has resulted in an estimated four to six million cases of CHIK fever with painful, often chronic, arthritides and an ongoing worldwide public health problem [3], [5]. The future occurrence of autochthonous cases in the mainland Americas seems inevitable with frequent travel-associated virus introduction, and the likelihood of a resulting outbreak is predicted to be high [6], [7]. Thus, the need to develop therapeutics and vaccine candidates for protection against this virus is ever more urgent. A cell culture-adapted LAV (181/25) is available as an investigational drug to at-risk researchers [8], [9]. However, insufficient attenuation and consequent reactogenicity problems have precluded its licensure for general use [8]. New strategies are required to develop CHIKV LAVs combining a more refined balance between attenuation and immunogenicity [10].

Like all members of the genus, CHIKV has an infectious, single-stranded RNA genome of ∼11 kb with a m^7^G 5′ cap structure and a 3′ polyadenylated tail (reviewed by [11]). Within this genome are encoded four non-structural (nsP1-4) and three structural (C, 6K/E1 and pE2) proteins from two open reading frames in the genomic and subgenomic RNAs, respectively [12], [13]. CHIKV virions have icosahedral symmetry, with a glycoprotein shell enclosing the viral membrane and nucleocapsid [14]. pE2 and E1 glycoproteins insert into the cell's endoplasmic reticulum as they are synthesized, forming heterodimers that trimerize into the viral ‘spikes’ and envelop nucleocapsids, budding as virus particles from the cell's plasma membrane [11]. pE2 is cleaved by host furin into E2 and a released E3 fragment during egress to produce the mature, fusion-competent virions in preparation for the next round of infection.

CHIKV isolates bind to cell surface receptors *via* E2 and fuse with cell membranes by clathrin-independent, Eps15-dependent, endocytosis *via* E1 [15]. The relationship between alphaviruses and their receptors is a complex one with many questions still unanswered and the identity of the receptor(s) utilized by wild-type isolates is still elusive with the exception of C-type lections, DC-SIGN and L-SIGN [16]. For many years, receptor identification was clouded by the use of strains that had adapted to growth in cultured cells. Fifteen years ago, we demonstrated that *in vitro* passage of the prototypic Old World alphavirus, Sindbis (SINV), in different laboratories had resulted in the accumulation of positively charged mutations in the E2 glycoprotein, which dramatically improved the virus-cell surface receptor interaction *in vitro* [17], [18]. In the converse experiment, Griffin and coworkers showed that *in vivo* passage in immune-deficient mice of a laboratory SINV strain could select for acquisition of negative charge and reduced heparan sulfate (HS)-dependence *in vitro* [19], [20]. Amino acid substitutions that increased net positive charge in certain E2 regions could dramatically increase per particle infectivity in cultured cells, dependent upon ionic interaction with negatively charged, cell-surface HS chains [17], [19], [21]. The following observations are of profound importance to the current study and to the biology of Old World alphaviruses in general: i) the substitution for positively charged residues in E2 that confer enhanced, HS-dependent infectivity *in vitro* is a common phenomenon amongst cell culture-passaged strains of SINV [17]–[19], [21], Ross River (RRV; [22]) and Semliki Forest (SFV; [23]) viruses; ii) these mutations can be selected within only a few serial passages *in vitro* [17]; and iii) viruses whose *in vitro* infectivity is enhanced by artificial HS attachment/entry are typically attenuated/avirulent *in vivo* from the natural infection route, at least in part due to reduced level and/or duration of viremia [20], [24].

We demonstrated recently that a wild-type CHIKV strain (LR2006 OPY1), isolated during the La Réunion outbreak and sequence-stabilized in cDNA clone form (CHIKV-LR; [25], [26]), exhibited no significant dependence upon HSPGs or other glycosaminoglycans (GAGs) for infectivity *in vitro* [27]. In contrast, the 181/25 CHIKV LAV candidate, derived by 18 serial passages of wild-type CHIKV (strain 15561) in MRC-5 fibroblasts to achieve attenuation [9], was highly dependent upon ionic interaction with HS for infectivity [27]. We predicted that this was due to an amino acid substitution at E2 position 82 [27], which was subsequently shown by Weaver and coworkers to attenuate both CHIKV-15561 and CHIKV-LR *in vivo* [28].

Here, we have exploited existing knowledge to deliberately select for and identify a set of E2 mutations that confer HS-dependence for infectivity by serial passage of wild-type CHIKV-LR on different cell-types *in vitro*. Single amino acid mutations that became predominant in the virus population within only five to ten passages through mammalian or mosquito cells were predicted by computational modeling to alter the electrostatic profile of the E2 glycoprotein and increase net positive charge in two exposed regions. By individual introduction of these mutations into CHIKV-LR, we have identified a panel of E2 mutations that confer reduced virulence in a murine model of musculoskeletal disease (MSD) and associated these with particular aspects of dependence upon HS for attachment/infectivity *in vitro*. In particular, we identified a novel mutation at E2 position 79 that increased attenuation over the E2-82R mutation present in the 181/25 LAV but did not diminish immunogenicity or protective efficacy. The positions of the attenuating mutations are clustered within two regions in the three-dimensional structure of the alphavirus trimer-heterodimer with the most attenuating mutated residues prominently exposed to the exterior of the spike. Furthermore, mutations conferring the greatest attenuation were associated with very small plaque size and sensitivity to competition with the HS analog, heparin. We propose this approach as an informed means to create mutant viruses and utilize *in vitro* HS interaction phenotypes and structural modeling to identify promising candidates for inclusion in CHIKV and other arboviral LAVs.

## Materials and Methods

### Ethical statement

This study was carried out in strict accordance with the recommendations in the Guide for the Care and Use of Laboratory Animals of the National Institutes of Health. All animal procedures were performed according to a protocol approved by the Institutional Animal Care and Use Committee of the University of Pittsburgh (Protocol 1001073).

### Mice

Pregnant and 21 d old CD-1 (Charles River Laboratories), and 8 wk old STAT129 (Taconic Laboratories) mice were housed under specific pathogen free conditions and all experiments were conducted at ABSL-3.

### Cell lines

BHK-21, CHOK1, pgsA745, pgsD677, and RAW264.7 cells were cultured as previously described [27], [29]. MC3T3-E1 osteoblasts were maintained in alpha minimum essential medium (AMEM) supplemented with 10% fetal bovine serum (FBS), 1 mM sodium pyruvate and 0.05 g/mL L-glutamine.

### *In vitro* passage

CHIKV-LR virus stock was serially passaged 10 times in triplicate series on CHOK1, pgsA745, or C6/36 cells, with a 1∶100 dilution of progeny virions between passages. At P5 and P10 supernatant from infected cells was placed in Tri Reagent-LS (MRC) containing 5 µg of tRNA carrier, and total RNA was extracted as per manufacture instructions. To sequence mutations in E2, cDNA was generated using RT-PCR (Roche) with a specific primer in the E1 gene, immediately downstream of the E2 gene 3′ terminus (GCAGCCTCTTGGTATGTGGC), and the entire pE2 gene was PCR amplified (S-CTAATGAAGGAGCCCGTACA; AS-GCAGCCTCTTGGTATGTGGC) using Deep Vent polymerase (NEB). The PCR fragment was either directly sequenced (Retrogen) or cloned into pCR-Blunt (Invitrogen) and sequenced.

### Viruses

pE2 gene mutations were introduced into the cDNA clone of CHIKV-LR using the Quick Change II XL mutagenesis kit (Stratagene). CHIKV-LR reporter viruses were created by inserting a cleavable in-frame fusion between capsid and E3 using Quick Change II XL mutagenesis to insert a PCR fragment at the capsid/E3 junction that encodes the first five amino acids of E3 fused in-frame with firefly luciferase (fLuc) followed by the 2A-like protease of *Thosea asigna* virus. Stocks of CHIKV-LR, E2 mutant viruses, and reporter viruses were generated from cDNA clones as previously described [27]. Briefly, cDNA was linearized and *in vitro* transcribed (mMessage mMachine, Ambion) to generate infectious, capped viral RNA genomes. Viral particles were harvested from supernatant of BHK cells 18–24 h post-electroporation with these RNAs. For all virus stocks, supernatant was clarified by centrifugation and single-use aliquots were stored at −80°C.

### Genome quantification

Virus stocks (200 µL) were treated with 40 U of RNase ONE (Promega) for 1 h at 37°C to remove any contaminating RNA that was not encapsulated in the virion and added to Tri Reagent-LS (MRC) along with 5 µg of carrier tRNA, before RNA was extracted per manufacture instructions. Equal total RNA concentration was used for reverse transcription (RT) with a primer complementary to sequence in the nsP2 gene and tagged with T7 to reduce background (5′-CGTAATACGACTCACTATAAGTACGTTGACGTGCTCTGACGTT-3′). Equal volumes of cDNA were used for real-time (q) PCR using SYBR green (Fermentas) to detect nsP2 on the positive strand (nsP2-TCfGTGTTAACGTGCTTCAGAGGGT; T7-GCGTAATACGACTCACTATA). A standard curve for viral genome number by qRT-PCR of RNA *in vitro*-transcribed from the CHIKV-LR cDNA clone. qRT-PCR results were analyzed using CFX Manager software (BioRad).

### Molecular modeling

The models of the E1/E2 trimer of the 05-115 CHIKV strain and mutants, including the E2 N-terminal tail that is missing in the CHIKV template trimer structure (pdb: 2XFB) [14], were constructed by using Modeller version 9v8 [30]. The presence of the N-terminal tail was found to have significant influence on the electrostatic potential in the areas of interest in the current studies. Charge of individual atoms and their radius parameters based on an amber force field [31] were generated by pdb2pqr program [32]. Electrostatic potential was generated by Adaptive Poisson-Boltzmann Solver (APBS) package [33]. A linearized Poisson-Boltzmann equation was applied with dielectric constant 2.0 for protein and 78.0 for solvent. Electrostatic potential on solvent accessible surface in the range from -5 kT to 5 kT and solvent radius 1.4 A was visualized with the PyMol Molecular Graphics System (PyMol Molecular Graphics System, Schrodinger, LLC).

### Morbidity and mortality studies

1 d old CD-1 mice were inoculated subcutaneously in the ventral thorax, 21 d CD-1 and 8 wk old STAT129 mice were inoculated subcutaneously in the hind footpad with either 10 µl of 10^5^ genomes or 10^3^ plaque forming units (PFU) of CHIKV viruses diluted in Optimem (Invitrogen). Mice inoculated with equal genome equivalents (GE) were inoculated with 10^5^ genomes (equivalent to ∼10^3^ PFU of CHIKV-LR). Mice were weighed and monitored daily for clinical signs of disease and mice showing severe signs of disease were monitored twice a day. The width and height of the metatarsal region of the rear footpad of CD-1 and STAT129 mice were measured daily with a caliper. Changes in footpad swelling were expressed as fold change in area (width×height) compared to pre-inoculation area. AST and percent mortality were calculated. Surviving mice were bled and challenged with 10^3^ PFU of CHIKV-LR 21 d post primary infection.

At 48 h post-infection (p.i.), groups of three mice were euthanized with isofluorane and then exsanguinated by cardiac puncture to collect blood. The serum was separated from the blood using Microtainer tubes (Becton-Dixon). Mice were then perfused with PBS-1% DBS virus diluent (VD). Tissues collected were homogenized in VD by mechanical disruption. Virus titer was assessed in supernatants from homogenized tissue by standard plaque assay on BHK cells and titers were expressed as PFU/g, mL or draining lymph node (DLN). Serum cytokine concentrations were measured using a mouse cytokine 20-plex kit (Invitrogen) per manufacture instructions and analyzed using the BioRad Bioplex 200.

### Antibody neutralization assay

Serum was diluted at a final concentration of 1∶20 in VD containing the CHIKV-LR reporter virus expressing fLuc. Serum and virus were incubated together at room temperature for 30 min before being used to infect a 96-well plate of BHK cells for 1 h at 37°C. Cells were washed twice with VD before culture media was added and the cells were then incubated for 16 h before cell lysates were harvested using passive lysis buffer (Promega). fLuc substrate (Promega) was added and relative light units (RLUs) were determined by microplate luminometer (Orion).

### Infectivity in salt

Virus was diluted in either RPMI1640 (has a basal salt level of 103 mM NaCl) or RPMI1640 containing different concentrations of NaCl and used to infect MC3T3-E1 cells for 1 h at 37°C before overlaying with immunodiffusion-grade agarose.

### Heparan sulfate dependency assays

#### Infectivity on pgsA675 and pgsD677 cells

CHOK1 (control cells), pgsA745 (GAG-deficient) and pgsD677 (HS-dependent) were infected with virus for 1 h at 37°C before overlaying with immunodiffusion-grade agarose (MP Biomedical). At 48 h p.i., cells were fixed overnight with 4% paraformaldehyde (PFA), PFA and overlay were removed, and cells were stained for CHIKV antigen as previously described [27].

#### Heparin assays

For the heparin competition assay, virus was diluted in Optimem (Invitrogen) either containing 200 µg/ml of heparin (Sigma) or BSA then incubated on ice for 30 min. Then MC3T3-E1 cells were infected on ice for 30 min to synchronize infection before overlaying with immunodiffusion-grade agarose was added for a standard plaque assay. To determine if heparin could inhibit plaque formation, MC3T3-E1 cells were infected for 1 h at 37°C before overlaying with immunodiffusion-grade agarose containing 200 µg/mL of either heparin or BSA. Plaque size was measured using a caliper.

#### Heparinase digestions

MC3T3-E1 cells were washed once with VD before incubation with various concentrations of Hep I, II, or III or VD for 1 h at 37°C. Cells were washed twice with VD and infected for 1 h at 37°C before overlaying with immunodiffusion-grade agarose for a standard plaque assay.

### Statistical analysis

For the footpad swelling, the area of under the curve was determined (GraphPad PRISM software) to assess the differences in swelling during the entire course of infection and then Student's t test was used to determine significance. Student's t test was used for all other experiments.

## Results

### *In vitro* passage of wild-type CHIKV-LR rapidly selects for positively charged mutations in the E2 glycoprotein

**Figure 1:**
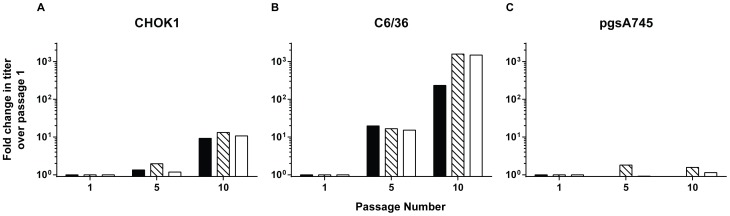
Cell culture passage of CHIKV-LR. CHIKV-LR virus stock was serially passaged 10 times in triplicate parallel series on: A) CHOK1; B) C6/36; or C) pgsA745 cells. After each amplification, the quantity of infectious virions was titered on BHK-21 fibroblasts by standard plaque assay. The fold-increase in titer at passage (P) 5 and P10 were normalized to the titer at P1. Each bar represents one individual passage series.

As a proof of concept, we began these studies by passaging CHIKV on cells from two evolutionarily divergent organisms, Chinese hamster ovary (CHO) K1 fibroblasts and C6/36 *Aedes albopictus* mosquito cells. A population of wild-type strain CHIKV-LR particles with maximal genome sequence homogeneity was generated by transfection of *in vitro*-transcribed, full-length, infectious viral RNA genomes into BHK-21 fibroblasts. This virus population was then subjected to a positive selection pressure for rapid growth on CHOK1 or C6/36 cells by performing ten sequential passages in triplicate parallel series. Infectious virion yields in supernatants, harvested after each amplification, declined slightly on both cell-types in the first two passages (data not shown) but then gradually increased to surpass passage 1 (P1) yields by ∼10 or 1,000-fold on mammalian or mosquito cells, respectively (Fig. 1). These data gave an initial indication of adaptation to cell culture within five to 10 passages. Importantly, infectious virion yields were not significantly enhanced by passage on CHOK1-derivative pgsA745 cells, deficient in the synthesis of GAG chains, suggestive that GAG-dependent changes underlay cell culture adaptation.

The sequence encoding the entire pE2 protein (E3 and E2) was analyzed from virus populations at P5 and P10 to identify mutations that might have accumulated during cell culture passage (Table 1). Each population of RT-PCR products was sequenced to reveal mutations present in the majority of packaged genomes. In addition, several individually cloned RT-PCR products from each P10 population were sequenced to determine whether amino acid substitutions were occurring alone or together, and to identify mutations occurring at lower frequency in the population. Passage on C6/36 mosquito cells selected strongly for an E to K substitution at E2 position 79, as this variant was the majority (or consensus) sequence for progeny virus genomes by P5 in three parallel passage series, and was retained through P10. In two of five individually cloned pE2 sequences from one passage series, we also identified a deletion of the codon for negatively charged E2-166E in mosquito cell-passaged virus populations. This mutation arose independently of E2-79K, which was found in the other three sequenced clones for this passage series, and in all sequenced clones for the two other passage series. Surprisingly, no other nucleotide mutations were detectable at this depth. Passage on CHOK1 fibroblasts selected strongly for an S to R substitution at E2-159 in the majority sequence in each of the three parallel passage series within five passages, and was retained as the dominant mutation in two passage series through P10. Interestingly, however, by P10 in the third passage series the dominant mutation in the population, and in four of six individual clones, was the E2-79 E to K substitution also selected on C6/36 cells. Three additional pE2 mutations were revealed in individual virion sequences. An E2-55 G to R substitution occurred at two out of five frequency in the second passage series on CHOK1 cells, while the combination of E2-99 H to Y/E2-168 E to K was present in two out of six of the population of the third passage series. After serial passage on GAG-deficient CHO cells, a dominant E2-264 V to A substitution was detected in one of three passage series at P5 and two of three at P10.

**Table 1:** Mutations identified in the E2 glycoprotein at passage 5 and 10 after cell culture adaptation of CHIKV-LR.

Virus	E2 amino acid position							
	55	79	82	99	159	166	168	264
**WT-LR**	G	E	G	H	S	E	E	V
**CHOK1 5.1**	G	E	G	H	**R**	E	E	V
**CHOK1 5.2**	G	E	G	H	**R**	E	E	V
**CHOK1 5.3**	G	E	G	H	**R**	E	E	V
**CHOK1 10.1**	G	E	G	H	**R**	E	E	V
**CHOK1 10.2**	G	E	G	H	**R**	E	E	V
**CHOK1 10.2a**	**R**	E	G	H	S	E	E	V
**CHOK1 10.3**	G	**K**	G	H	S	E	E	V
**CHOK1 10.3a**	G	E	G	**Y**	S	E	**K**	V
**C6/36 5.1**	G	**K**	G	H	S	E	E	V
**C6/36 5.2**	G	**K**	G	H	S	E	E	V
**C6/36 5.3**	G	**K**	G	H	S	E	E	V
**C6/36 10.1**	G	**K**	G	H	S	E	E	V
**C6/36 10.2**	G	**K**	G	H	S	E	E	V
**C6/36 10.3**	G	**K**	G	H	S	E	E	V
**C6/36 10.3a**	G	E	G	H	S	**-**	E	V
**pgsA745 5.1**	G	E	G	H	S	E	E	V
**pgsA745 5.2**	G	E	G	H	S	E	E	**A**
**pgsA745 5.3**	G	E	G	H	S	E	E	V
**pgsA745 10.1**	G	E	G	H	S	E	E	V
**pgsA745 10.2**	G	E	G	H	S	E	E	**A**
**pgsA745 10.3**	G	E	G	H	S	E	E	**A**

Suffix ‘a’ indicates mutations identified only in individual viruses but not in the majority population.

### E2 glycoprotein electrostatic potential is altered by the cell culture-adaptive mutations

In total, seven amino acid changes were identified in the E2 proteins of the cell culture-passaged virus populations, four of which substituted a neutrally, or negatively, charged residue with a positively charged one (G to R at E2-55; E to K at E2-79; S to R at E2-159 and E to K at E2-168), while a fifth deleted a negatively charged amino acid (E2-Δ166E). Neither the V to A substitution at E2-264 nor the H to Y substitution at E2-99 would be anticipated to alter the net charge.

**Figure 2:**
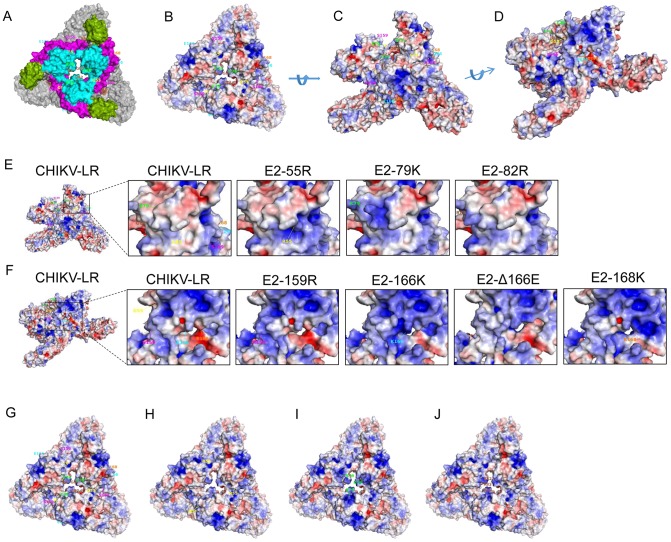
Effects of positively charged amino acid substitutions in the CHIKV E2 glycoprotein on electrostatic potential in the E1/E2 trimer-heterodimeric spike. The trimeric CHIKV-LR E1/E2 heterodimer was modeled and APBS was used to determine the electrostatic potential of the CHIKV-LR E2 protein, the location of mutated residues and predicted changes to the electrostatic potential conferred by these mutations. (A) Top view of the CHIKV-LR trimer-heterodimer with E1 shown in gray, E2 domain A in cyan, domain B in green, and domain C in brown. (B–D) The electrostatic potential of the CHIKV-LR trimer-heterodimer where blue indicates positive charge, white indicates neutral charge and red indicates negative charge. B) top view; C) side view I; and D) side view II. (E & F) For ease of viewing, the region of the E2 molecule altered by each mutation is magnified showing only one of the E2 molecules in the trimer-heterodimer. E) Magnification from side view I of the “wing” portion of domain A that contains the E2-55R, E2-79K and E2-82R mutations; F) Magnification of side view II of the acid sensitive region in arch 1 between domains A and B that contains the E2-159R, E2-Δ166E/166K and E2-168K mutations. G–J) The top view of the electrostatic potential of trimetric heterodimer for the E2 mutations in the “wing” portion of domain A: G) CHIKV-LR, H) E2-55R, I) E2-79K and J) E2-82R.

Based upon the X-ray crystallographic E1/E2 heterotrimeric structure of the CHIKV clinical isolate 05-115 [14], [34], we generated a 3D structural model of the trimeric envelope glycoprotein heterodimer (Fig. 2A) and used Adaptive Poisson-Boltzmann Solver (APBS; [33]) to determine the electrostatic potentials for the trimer-heterodimers of the wild-type CHIKV-LR strain and the identified mutations (Fig. 2B–D). The cell culture passage-selected E2 mutations mapped to two regions previously defined by Voss *et al.* [14]. Residues E2-55 and E2-79 mapped to the “wing” portion of Domain A in the region of insertion strands i3, i5 and i6 into Ig-like domains (Fig. 2C). The E2-82R mutation selected during 18 serial passages of the 15561 wild-type CHIKV strain on MRC-5 fibroblasts to produce the 181/25 CHIKV LAV candidate [35], and previously shown to attenuate both 15661 and CHIKV-LR [28], also mapped within this region. When viewed from above the three-fold axis of symmetry (Fig. 2B), the E2-55 residue resides in a cleft, overhung by other portions of Domain A, but facing outward from the spike interior adjacent to the β ribbon connector, whereas the E2-79 and E2-82 residues lie toward the apical surface of the protein, facing the solvent-exposed interior of the E1/E2 heterotrimer, with E2-79 more exposed than E2-82.

The E2-159R, E2-Δ166E and E2-168K mutations mapped to the acid-sensitive region in arch 1 of the β ribbon connector between Domains A and B (Fig. 2D). Interestingly, an E to K substitution at E2-166 was previously selected during passage of CHIKV-06.049 on human epithelial carcinoma HeLa cells [36]. The E2-159, E2-166 and E2-168 residues lie within the β ribbon connector facing outward from the heterotrimeric spike interior essentially on the opposite side of the E1/E2 heterodimer from E2-79 and E2-82 residues. E2-166 and E2-168 residues are close to areas of contact between the β connector and E1, although residue E2-166 is located more towards the heterotrimer exterior than E2-168 which is deeper within an invagination of the spike between the β connector and E1. The E2-159 residue lies higher and more towards the cleft in Domain A, where E2-55 is located.

For ease of viewing, regions affected by the changes in electrostatic potential created by each mutation are shown magnified on only one of the E2 molecules in the heterotrimer (Fig. 2E & F). Those amino acid mutations that were predicted to increase net positive charge also produced localized increases in the computer-predicted positive electrostatic potential on the surface of the E2 protein. In contrast, the E2-Δ166E deletion mutation appeared to affect a broader area and, at the resolution of the model, alter contact regions between the β connector and E1. The prominent exposure of the positive-charge shift conferred by the highly attenuating E2-79K mutation in comparison with the similarly located E2-55R and E2-82R is particularly apparent in the top view of the spike three-fold axis of symmetry (Figs. 2H–J).

### Positively charged amino acid substitutions in E2 result in a range of HS dependencies

Each amino acid substitution or deletion discussed above, including E2-82R and E2-166K, was introduced separately into the CHIKV-LR cDNA clone. Stocks of CHIKV-LR and the E2 mutant viruses were generated by transfection of *in vitro*-transcribed, capped genomes, and not passaged further. Reasoning that the positive electrostatic potential increases in E2 would result in a dependency upon cell surface HS for infectivity, we compared particle infectivity on CHOK1 cells *versus* derivatives that lack the ability to synthesize either all GAG chains (pgsA745) or just HS (pgsD677; [37]). Like other Old World alphaviruses, the infectivity of wild-type CHIKV-LR did not depend upon the presence of these sulfated glycans (Supplemental Fig. S1; [27], [38]). In contrast, the infectivities of LR-55R, LR-79K, LR-82R, LR-159R, LR-166K, LR-Δ166E and LR-168K for CHO cells all exhibited significant dependence upon GAGs, and this phenotype was almost completely conferred by the absence of HS alone. Neither E2-99Y, nor E2-264A mutation, exhibited significant dependence upon GAGs for infectivity (data not shown). In our experience the usefulness of the pgsD677 and pgsA745 CHO cells is limited to determining whether or not viral infectivity is affected by the absence of HS or GAGs but does not accurately determine relative degrees of dependency. However, these data indicate that predicted increase in exposed positive charge on E2 in the trimer-heterodimeric spike correlates with a significant dependence upon HS for infection of CHO cells. Most of the mutations increased per-particle infectivity, with the notable exceptions of E2-Δ166E and E2-159R (data not shown), which reduced infectivity unless HS was present, indicating that the latter mutations may compromise attachment/entry *via* another receptor(s) pathway used by CHIKV-LR.

### Positively charged amino acid substitutions in E2 confer a range of virulence phenotypes *in vivo*

Focusing upon those mutations that increase electrostatic potential on the E2 surface positive charge compared to wild-type CHIKV-LR (Fig. 2), and conferred the ability to bind HS (Supplemental Fig. S1), the virulence of each mutant relative to wild-type CHIKV-LR was assessed in a murine model of MSD with edema/inflammation, by measuring hind-limb swelling across the metatarsal region after subcutaneous inoculation of virus into the footpad, as described previously [27], [39], [40].

**Figure 3:**
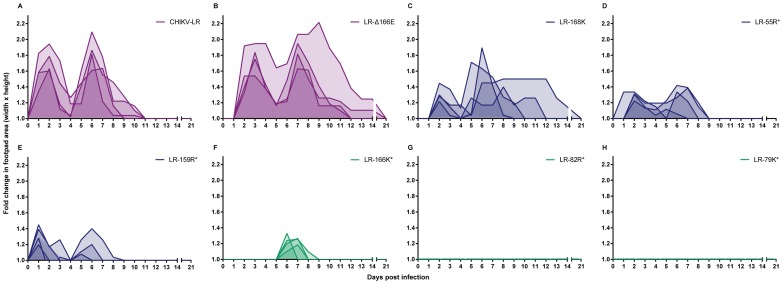
Musculoskeletal disease following subcutaneous infection of CD-1 mice with E2 mutant viruses. 21-1 mice were infected subcutaneously in the left rear footpad with 10^5^ GE (normalized to 10^3^ PFU of CHIKV-LR) and the metatarsal region (height and width) was measured daily; fold-change over day 0 was calculated based upon pre-infection footpad area. Viruses are color-coded based on disease phenotype: (A–B) hind-limb swelling with little or no attenuation (purple; (A) CHIKV-LR and (B) LR- Δ166E), (C–E) partial attenuation (blue; (C) LR-168K, (D) LR-55R and (E) LR-159R) and (F–H) highly attenuated with little or no hind-limb swelling (green; (F) LR-166K, (G) LR-82R and (H) LR-79K) compared to CHIKV-LR. Individual mice are graphed with the intensity of shading indicating the proportion of mice with overlapping levels of hind-limb swelling. *p<0.001 compared to CHIKV-LR.

Two waves of limb swelling were consistently observed in mice infected with CHIKV-LR, the first peaking 1–2 d p.i., and the second 6–7 d p.i., with complete resolution by 12–14 d p.i. (Fig. 3A). Varying degrees and patterns of attenuation compared to the wild-type virus were observed for the mutant viruses, which we grouped into three categories for clarity. In the first category (purple), little or no attenuation was observed for LR-Δ166E infection (Fig. 3B) compared to wild-type CHIKV-LR, although the onset of clinical signs was delayed by ∼24 h and the duration of disease was longer for some animals. In the second category (blue), partial attenuation was observed for several virus mutants (Fig. 3C–E). LR-168K (Fig. 3C) was ∼24 h delayed and attenuated at 1–2 d p.i. but produced wild-type levels of swelling in some cases in the second phase. Interestingly, some animals infected with LR-168K also exhibited a more prolonged swelling than we observed for the wild-type virus infection. LR-55R (Fig. 3D) and LR-159R (Fig. 3E) were not delayed but demonstrated significantly reduced swelling compared with CHIKV-LR in both waves. In the third category (green), three virus mutants were highly attenuated, causing little or no hind-limb swelling (Fig. 3F–H). LR-166K (Fig. 3F) caused a transient, mild swelling only in the second wave, whereas no evidence of swelling was detectable for LR-79K (Fig. 3G) or LR-82R (Fig. 3H). This categorization and color scheme is used for subsequent figures to demonstrate prominent associations of genotype and phenotype.

**Figure 4:**
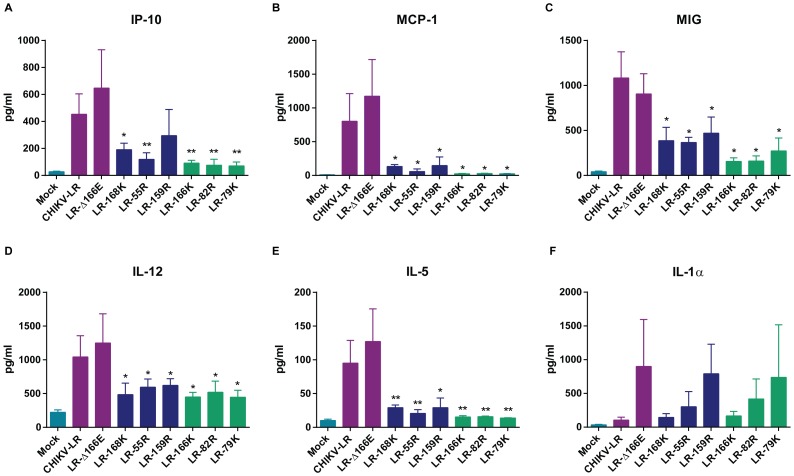
Cytokine induction following subcutaneous inoculation of CHIKV E2 mutant viruses. 21-1 mice were infected subcutaneously in the left rear footpad with 10^5^ GE. At 48 h p.i. sera were collected and analyzed for cytokine/chemokine induction using a 20-plex luminex bead kit. A) IP-10; B) MCP-1; C) MIG; D) IL-12; E) IL-5; F) IL-1α. Viruses are color-coded based on MSD severity: little or no attenuation (purple), partial attenuation (blue) or highly attenuated (green) compared to CHIKV-LR. Error bars represent standard deviation. *p<0.05 and **p<0.01 compared to CHIKV-LR.

A number of chemokines and cytokines have been associated with the acute phase of disease in humans, including MIG (CXCL9), MCP-1 (CCL2) and IP-10 (CXCL10) [41]–[45]. Furthermore, in a murine model similar to the one used here, MCP-1 has been shown to play an important role in pathogenesis of CHIKV-induced MSD [39], [41]. Interestingly, comparison of inflammatory responses to CHIKV-LR and the E2 mutant viruses revealed that higher induction of two cytokines (IL-12 p35/p40 and IL-5) and three chemokines (MCP-1, MIG and IP-10) correlated well with the severity of MSD (Fig. 4). On the other hand, IL-1α, IFN-γ and IL-2 were significantly induced in all virus-infected animals at 48 h p.i. *versus* mock-infected counterparts but no association between disease severity and these cytokine levels was observed (Fig. 4 and Supplemental Table S1). No significant elevation of other measured cytokines was observed over mock-infected animals for any of the viruses during this acute phase of infection (Supplemental Table S1). These data not only indicate that the levels of certain inflammatory molecules provide early biomarkers of MSD severity or attenuation in mice, but further validate the murine model of human infection.

### Positive charge E2 mutations tend to increase specific infectivity of virus particles and infectivity was dependent on ionic interactions *in vitro*

**Figure 5:**
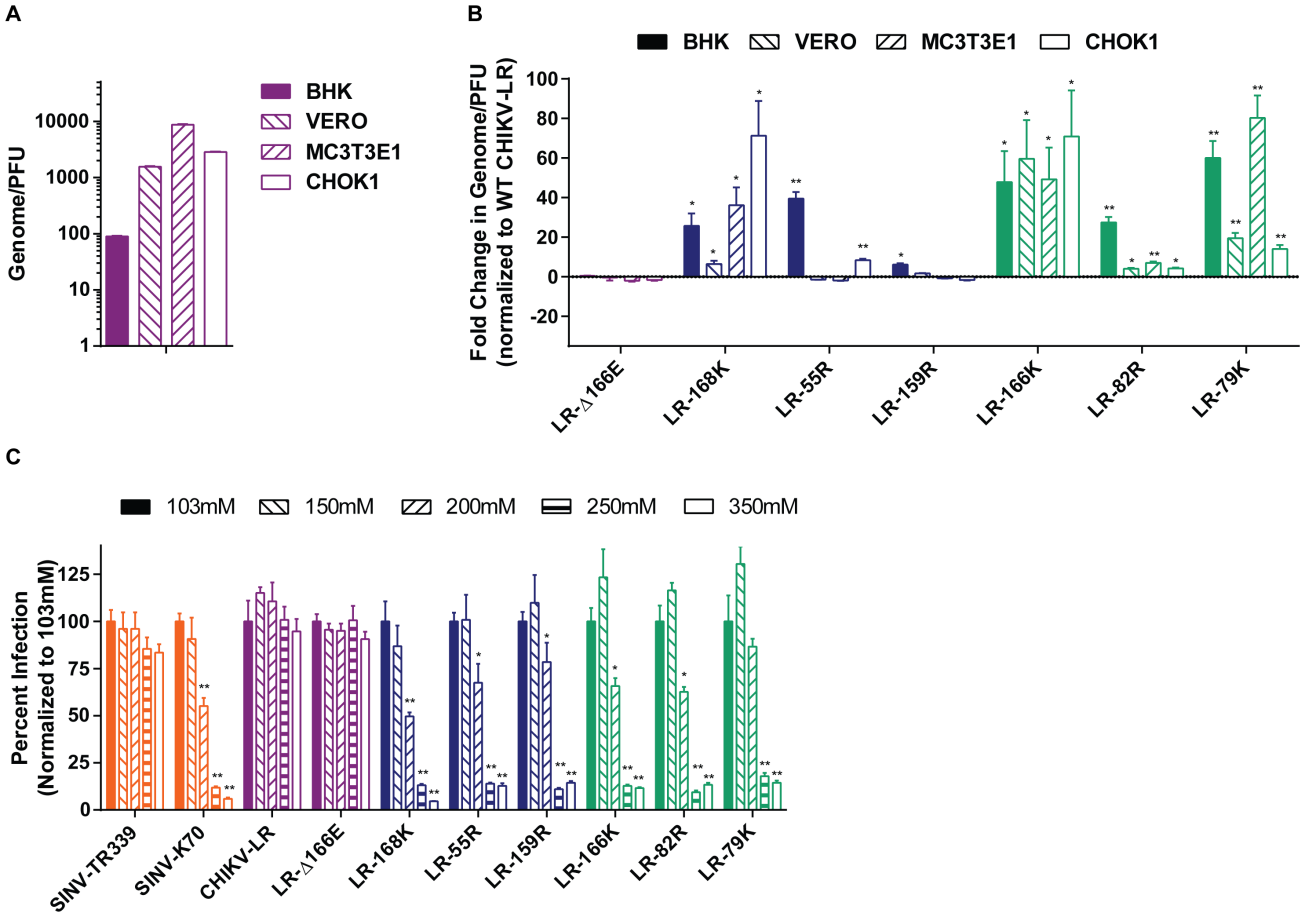
Positive charge CHIKV E2 mutations increase specific infectivity and infectivity is dependent on ionic interactions. Specific infectivity (GE/PFU) of A) CHIKV-LR WT; and B) the mutants CHIKV E2 mutants were evaluated on different cell types. For the CHIKV E2 mutants, fold-change in specific infectivity was normalized to CHIKV-LR. C) CHIKV E2 mutants were evaluated for dependency on ionic interaction for infectivity by interacting virus in either RPMI1640 (basal salt concentration of 103 mM NaCl) or RPMI1640 with increasing salt concentration and MC3T3-E1 cells were then infected for a standard plaque assay. Percent infectivity was normalized to 103 mM NaCl, which was set to 100%. Viruses are color-coded based on control groups (orange) or MSD severity: little or no attenuation (purple), partial attenuation (blue) or highly attenuated (green) compared to CHIKV-LR. Error bars represent standard deviation. *p<0.01 and **p<0.001 compared to CHIKV-LR (B) or 103 mM NaCl (C).

In an effort to identify particular *in vitro* phenotypes that could be used to identify promising mutations for a LAV, we compared multiple characteristics of the dependence of infectivity of each mutant upon HS. By calculating plaques per GE (specific infectivity), we estimated that under plaque assay conditions, ∼1% of wild-type CHIKV-LR virus particles initiated infection on BHK-21 fibroblasts, 0.1% on Vero cells, 0.05% on CHOK1 cells and 0.01% on MC3T3-E1 osteoblasts (Fig. 5A). These susceptibility differences may be due to reduced attachment, entry and/or to subsequent steps in propagation. The LR-Δ166E mutant exhibited minimal changes in specific infectivity on the four cell-types compared to wild-type CHIKV-LR. Otherwise, with a few exceptions (E2-55R on Vero and MC3T3-E1, and E2-159R on CHOK1 and MC3T3-E1), the cell culture passage-derived E2 mutations tended to increase the infectivity of CHIKV particles by up to 70-fold (Fig. 5B) and thus provided an advantage to virions in culture conditions. However, these data also indicated that the virus-host receptor interactions differ between cell-types, as the hierarchies of cell susceptibility and virion infectivity were not consistently maintained.

To further explore *in vitro* phenotypes we focused upon the MC3T3-E1 osteoblasts as a cell-type with relevance to CHIKV-induced MSD *in vivo* [46] and known to secrete extracellular matrix components including HSPGs [47]. Virus inocula were prepared in media with a range of salt concentrations to disrupt potential ionic interactions between the virus particles and cell-surfaces during the infection period. As expected from our prior studies, the infectivities of wild-type SINV (SINV-TR339) and CHIKV-LR particles exhibited no significant dependence upon ionic interaction, whereas an E2-70K mutation in SINV-TR339 (SINV-K70) makes this virus highly sensitive to ionic interactions [17], [21], [27]. Interestingly, although the electrostatic model of the E2 protein predicted that the deletion of residue E2-166E would increase net-positive charge, the infectivity of LR-Δ166E virus for MC3T3-E1 osteoblasts was insensitive to even the highest salt concentration (350 mM; Fig. 5C), and indistinguishable from wild-type CHIKV-LR by this assay. The infectivity of the other viruses with positively charged E2 amino acid mutations was significantly disrupted at 200–250 mM salt concentration (Fig. 5C), indicating that the vast majority of virions now relied upon an ionic interaction for infectivity.

### Increased infectivity tends to be conferred by ionic interactions, sensitive to blocking by heparin

**Figure 6:**
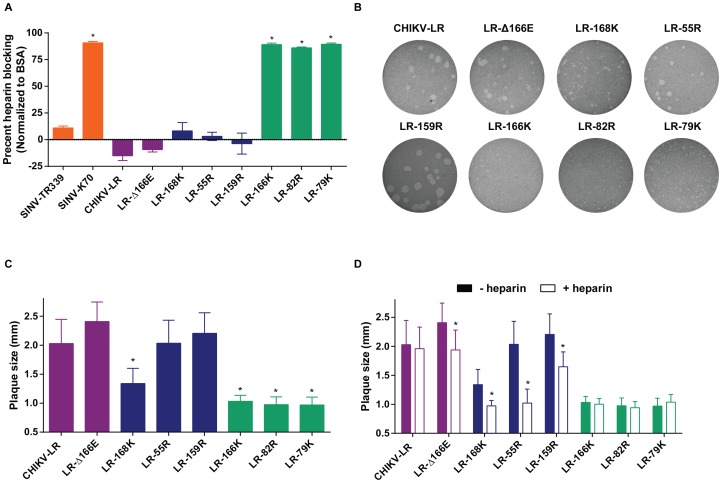
Soluble heparin can block infectivity and alter plaque size of several of the positive charge CHIKV E2 mutants on MC3T3-E1 osteoblasts. A) Soluble heparin was used to block infectivity of the virus by competing with the ability of the virus to attach to cells. Virus was incubated with 200 µg of either soluble heparin or BSA, infection was synchronized and overlay was added for a standard plaque assay. Percent heparin blocking was normalized to BSA control. B & C) Standard plaque assay on MC3T3-E1 was used to determine plaque size of the different CHIKV viruses. C) Plaque sizes were quantified by measuring the size of the plaques using a caliper. D) Virus was titered using a standard plaque assay on MC3T3-E1 cells in the presence or absence of 200 µg/mL of soluble heparin in the overlay. Plaque sizes were quantified by measuring the size of the plaques using a caliper. Viruses are color-coded based on control groups (orange) or MSD severity: little or no attenuation (purple), partial attenuation (blue), or highly attenuated (green) compared to CHIKV-LR. Error bars represent standard deviation. *p<0.001 compared to CHIKV-LR (B) or 103 mM NaCl (C).

When we examined the ability of a high concentration of soluble heparin (200 µg/mL) to block infection of MC3T3-E1 osteoblasts (Fig. 6A), we were surprised to discover that only LR-79K, LR-82R and LR-166K were effectively competed *versus* BSA-treated controls and this treatment reduced infectivity by >90%. However, heparin is a uniformly highly sulfated GAG, lacking the diversity of HS chain charge distributions and therefore does not necessarily block all HS-ligand interactions. It should also be noted that, because the soluble heparin treatment unexpectedly increased the infectivity of wild-type CHIKV-LR on MC3T3-E1 cells by ∼15%, all of the mutations except for E2-Δ166E significantly increased sensitivity to heparin blocking even if only by a small fraction. Compared to previous observations for other alphaviruses that ionic interactions were almost completely HS-mediated and competed by soluble heparin [17], [19], [20], [29], [48], these findings suggest a more complicated interaction for these CHIKV mutants.

While performing the above experiments, it was noted that plaque sizes on MC3T3-E1 osteoblasts were highly variable between mutants (Fig. 6B). When quantified and compared with CHIKV-LR (Fig. 6C), it was revealed that the plaques formed by LR-166K, LR-82R and LR-79K were extremely small, LR-168K plaques were of intermediate size, while the plaque sizes of LR-Δ166E, LR-55R and LR-159R did not differ significantly from wild-type virus. The heparin blocking assay measures only the ability of the virus to initiate infection in the presence of varying amounts of heparin. As an extension to this assay, we determined whether or not the addition of heparin to the overlay impacted virus plaque size phenotypes as a more sensitive measurement of HS-dependence. As expected the plaque size of the wild-type virus was not affected (Fig. 6D). The three viruses exhibiting the smallest plaque size were also unaffected by added heparin, presumably because cell-surface or extracellular matrix HS already inhibits the spread of these highly sensitive mutants. However, heparin reduced the plaque sizes of LR-55R and LR-168K to a size comparable with LR-79K, LR-82R and LR-166K, and reduced the plaque size of LR-159R and LR-Δ166E, significantly. The reduced plaque size of LR-Δ166E under heparin overlay along with the reduced infectivity on HS-deficient CHO cells (Supplemental Fig. S1) suggested that this virus has a slight dependence on HS depending upon culture conditions, which fit with the prediction of an alteration of the positive electrostatic potential.

### Increased infectivity conferred by positively charged E2 mutations tends to be disrupted by HS digestion from cell surfaces

**Figure 7:**
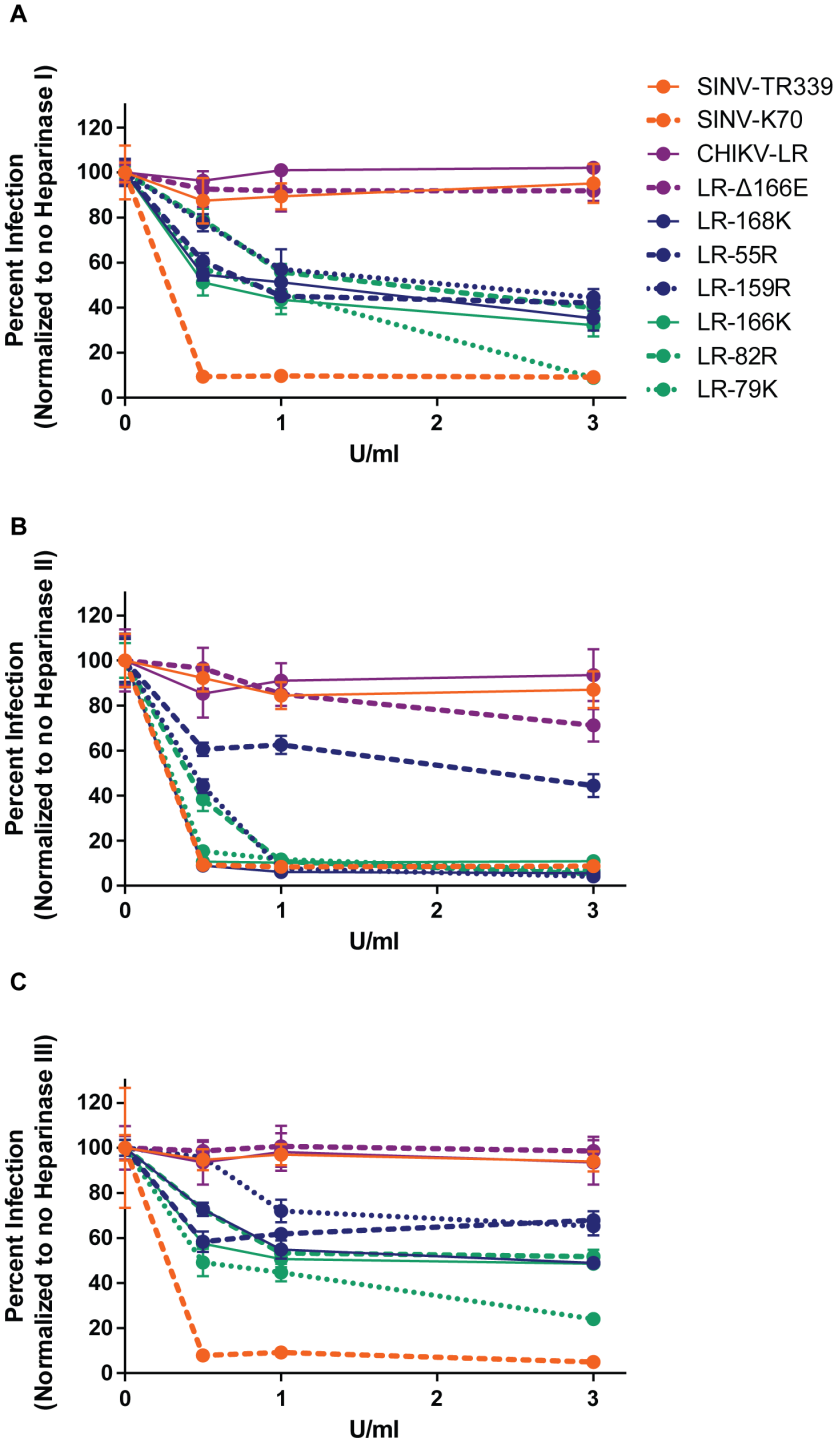
HS-dependent infectivity of the CHIKV E2 mutants by removal of HS on MC3T3-E1 cells. CHIKV E2 mutants were evaluated for dependency on HS for infectivity on MC3T3-E1 cells by digestion of HS with different heparinases (Hep). MC3T3-E1 cell surfaces were mock-treated, or digested with various concentrations of A) Hep I; B) Hep II; or C) Hep III and then infected with virus for a standard plaque assay on MC3T3-E1 cells. Percent infection was normalized to mock treatment which was set to 100%. Viruses are color-coded based on control groups (orange) or MSD severity: little or no attenuation (purple), partial attenuation (blue), highly attenuated (green) compared to CHIKV-LR. Error bars represent standard deviation. For all heparinases at all concentrations, there was no significant difference in infectivity for SINV-TR339, CHIKV-LR and LR-Δ166E compared to the no Hep control. For all of the CHIKV E2 mutants, with the exception of LR-159R at 0.5 U/mL of Hep III, had a significant decrease in infectivity compared to the mock-treated control (*p<0.01*).

We examined the infectivity of each virus for MC3T3-E1 cells digested with microbial heparinases, (Hep) I, II and III, which digest cell-surface HS chains (recently reviewed in [49]). Hep II acts with little specificity, cleaving both HS chains and heparin regardless of sulfation. In contrast, Hep I cleaves primarily heparin and highly sulfated HS regions, while Hep III primarily cleaves less-sulfated regions of HS chains. Thus, the HS structures remaining on the cell surface after digestion differ between heparinases. Successful digestion of HS was confirmed by the greatly reduced infectivity of SINV-K70. As expected, viruses with little infectivity dependence upon HS (SINV-TR339, CHIKV-LR and LR-Δ166E) were unaffected by the digestion of HS chains with Hep I, II or III, even when high concentrations of enzyme were used (Fig. 7A–C). With the exception of LR-55R, heparin blocking (Fig. 6A) correlated with >90% reduction in infectivity on MC3T3-E1 cells digested with Hep II. However, the infectivities of these viruses were only reduced ∼50% by digestion with Hep I or III suggesting that they were able to utilize residual chains for infection regardless of their sulfation level, unlike SINV-K70. The infectivity of LR-55R was reproducibly reduced by only ∼50% following digestion with Hep I, II or III. Overall, LR-79K infectivity appeared to be the most sensitive to the digestion of HS, followed by LR-166K.

### Attenuation of E2 mutants *in vivo* correlates with decreased spread from site of inoculation

**Figure 8:**
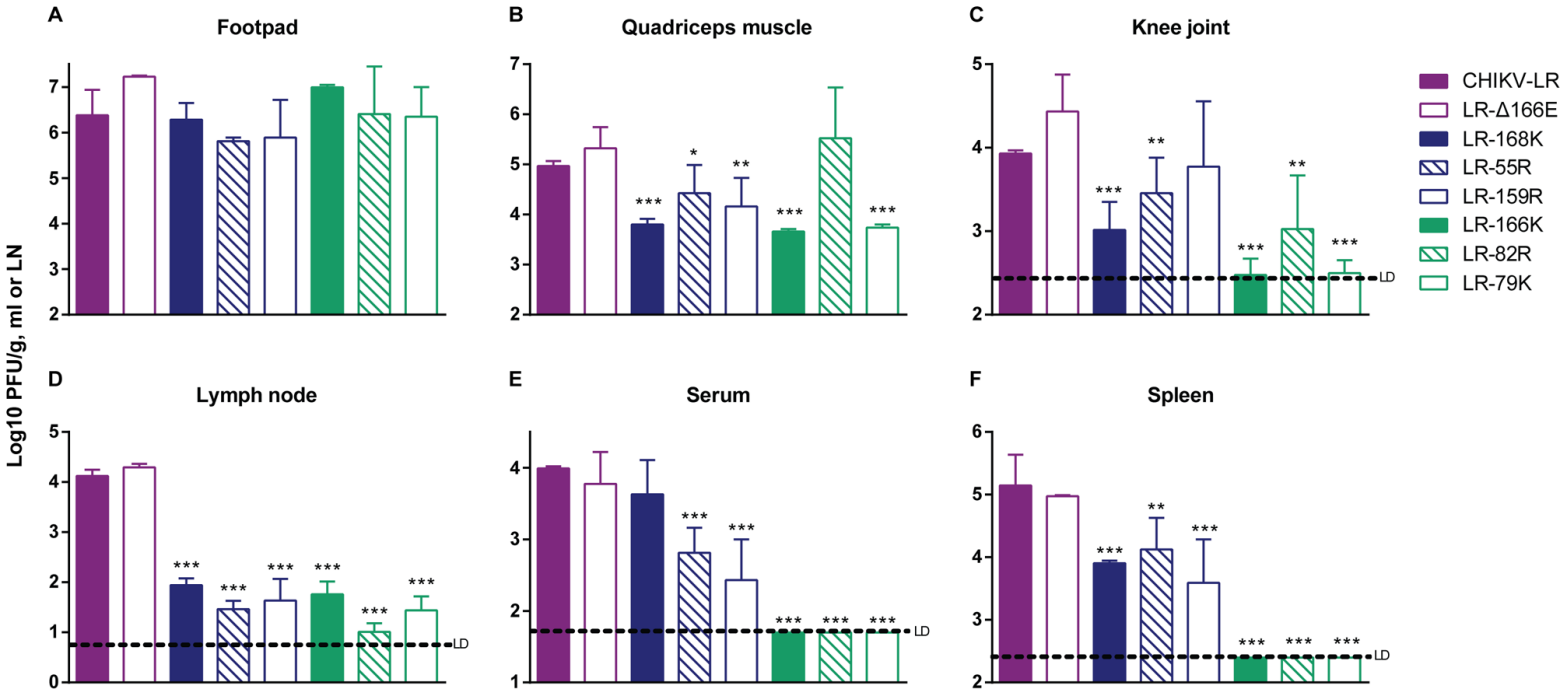
Viral load following subcutaneous inoculation of CHIKV E2 mutants. 21-1 mice were infected subcutaneously in the left rear footpad with 10^5^ GE. At 48 h p.i., A) footpad; B) quadriceps muscle; C) knee joint; D) popliteal lymph node (LN); E) serum; and F) spleen were harvested and analyzed for viral load by standard plaque assay. The limit of detection (LD) per g, mL, or LN is indicated. Viruses are color-coded based on MSD severity: little or no attenuation (purple), partial attenuation (blue) or highly attenuated (green) compared to CHIKV-LR. Error bars represent standard deviation. *p<0.05, **p<0.01 and ***p<0.001 compared to CHIKV-LR.

To better understand the reasons for attenuation of the HS-binding E2 mutants *in vivo*, especially LR-79K and LR-82R, we measured viral load in various tissues at 48 h p.i. (Fig. 8). This time-point was chosen to represent the first peak of swelling, and the previously observed peak of CHIKV-LR replication in this model [27].

#### Site of inoculation and musculoskeletal tissues

With the exception of LR-55R, viral titers at the inoculation site were similar to or higher than those of wild-type virus suggesting that the different E2 mutants were all capable of initiating replication in the skin (Fig. 8A). Viral loads for the attenuated E2 mutants were generally lower in MSTs than wild-type CHIKV-LR (Fig. 8B & C), whereas the LR-Δ166E mutant titers were comparable to wild-type, and LR-159R infection produced similar viral load in the knee joint as CHIKV-LR in keeping with the observed hind limb swelling. However, it was surprising to discover that LR-82R replicated to similar titer in the quadriceps muscle as the wild-type virus since this mutant caused no detectable MSD.

#### DLN, serum viremia and spleen

All of the attenuated viruses (groups 2 and 3) exhibited a highly significant inability to amplify within the DLN (Fig. 8D), suggesting a reduced or delayed spread either within the lymphatic vessels to the DLN or within the organ itself. With the exception of LR-168K, the viremic titers were also significantly reduced or undetectable in the cases of LR-166K, LR-82R and LR-79K (Fig. 8E). Notably, there was no detectable presence of the three most attenuated viruses, LR-79K, LR-82R, and LR-166K, in the spleen either likely due to the absence of viremic spread by this time p.i. (Fig. 8F). These data suggest that the attenuated E2 mutants are impaired to differing degrees in their ability to spread from the site of inoculation with a high degree of association with MSD severity. It is interesting, however, that viral loads in lymphoid tissues and serum viremia appear to correlate better with MSD and cytokine/chemokine levels than MST titers.

### Attenuation of E2 mutants in an immunocompromised mouse models

Based upon the studies above, we proposed that two of the mutant viruses were sufficiently attenuated for inclusion in LAV vaccine formulations: LR-82R and LR-79K. To stringently determine the degree and stability of their attenuated phenotypes, we infected more susceptible animals. We have previously shown that mice deficient in STAT1-dependent type I IFN signaling pathways had exacerbated MSD, whereas CHIKV-181/25 remained partially attenuated in these animals [27]. STAT1-deficient mice infected with LR-82R exhibited exacerbated hind limb swelling compared to 129/Sv control animals, similar to that caused by wild-type CHIKV-LR (Supplemental Fig. S2). However, LR-79K infection of the STAT1-deficient mice caused only mild MSD compared to CHIKV-LR and LR-82R, from which we infer that LR-79K is substantially more attenuated for ability to cause MSD than LR-82R or even the 181/25 LAV. Both LR-82R and LR-79K infections were lethal to the STAT1-deficient mice, however, supporting our prior contention that MSD and fatality are not closely linked.

### LR-79K and LR-82R elicit protective immunity and neutralizing antibodies

**Figure 9:**
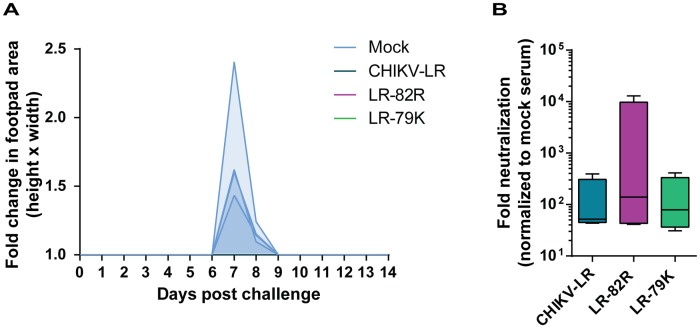
Protective immunity and neutralizing antibody elicited by LR-82R and LR-79K. 21-1 mice were infected with 10^5^ GE of CHIKV-LR WT, LR-82R, or LR-79K subcutaneously in the rear footpad. At 21 d p.i., mice were bled and challenged with 10^3^ PFU of CHIKV-LR. A) Hind-limb swelling post-challenge was determined by measuring the metatarsal region (height and width) daily and fold change was calculated based on pre-challenge footpad area. The legend indicates the virus used for the primary infection. Individual mice are graphed with the intensity of shading indicating the proportion of mice with overlapping levels of hind-limb swelling. B) Neutralizing antibody levels were determined by interacting a CHIKV-LR luciferase-expressing virus with sera and then infecting cells overnight. Lysates were then harvested and analyzed for luciferase signal. Levels of neutralizing antibody were determined as fold reduction in luciferase signal compared to mock sera. Error bars represent standard deviation.

Finally, we immunized CD-1 mice with LR-82R or LR-79K, and challenged with CHIKV-LR three weeks p.i., to determine whether or not these attenuated E2 mutants could efficiently elicit a protective adaptive immune response. All of the immunized mice were completely protected from development of MSD upon CHIKV-LR challenge, whereas mock-immunized mice developed mild MSD (Fig. 9A). Only the second wave of swelling was observed in these control animals most likely due to the ongoing age-dependent attenuation of CHIK-LR-induced MSD in this model. Protection from disease was coincident with the presence of neutralizing antibodies prior to challenge (Fig. 9B) and, although we have not shown directly that this confers the immunity, antibody responses have been shown to protect [50], [51]. Interestingly, all of the E2 mutants elicited levels of neutralizing antibody not dissimilar to the wild-type infection by 21 d p.i. (Fig. 9B and data not shown), despite there being highly significant differences in dissemination, replication and disease.

## Discussion

The gold standards for a successful LAV against mosquito-borne virus infection are the 17D strains of yellow fever virus (YFV), currently used for routine immunization where YFV is endemic and in regional mass vaccination campaigns at the first sign of an outbreak [52]. The 17D vaccines immunize 99% of vaccinees with only one inoculum dose, provide immune memory for decades, and very rarely cause vaccine-associated adverse events. The original 17D virus was fortuitously selected by extensive blind cell culture-passage in the 1930's [53]. Although, the molecular mechanisms underlying the consistent immunogenicity and stable attenuation of 17D are largely unknown and cannot yet be intentionally duplicated for viruses or even for YFV, increased HS interaction has been identified as significantly contributing to the attenuation of 17D [54]. Alphavirus LAV candidates have been generated for VEEV (TC83; [55]) and CHIKV (181/25; [9]) by serial passage in cell culture but both have encountered problems during clinical trial for reasons of inadequate immunogenicity in some vaccinees and residual virulence in others. Yet, both of these vaccines are attenuated, at least in part due to more efficient interaction with HS than their wild-type counterparts [27], [48]. Attaining the ideal balance between attenuation of disease and immunogenicity is extremely challenging, and to do so by design will require knowledge of virus biology and the optimization of rational attenuation strategies that can be combined to produce a safe and effective vaccine. Thus, one characteristic of most, if not all, arbovirus LAV is increased HS interaction over the wild-type virus, yet these mutations have previously been selected at random as a component of extensive blind passages without knowledge of their contribution to attenuation of the vaccine.

Our current studies represent the first attempt to evaluate systematically selected mutations that confer interaction with HS and attenuate CHIKV *in vivo*. We began by assuming that a rational method for identification of a panel of E2 glycoprotein mutants highly enriched in HS binding mutations would be serial passage of CHIKV virus on two evolutionarily divergent cell types. Within an organism, HS biosynthesis is primarily regulated by domain distribution and degree of sulfation (i.e., the distribution of *N*-substituents and the levels of 2-*O*- and 6-*O*-sulfation) resulting in different composition of HS species from different cellular or tissue sources [56], [57]. Furthermore, invertebrate HS has a number of unique properties, including unusually low *O-*sulfation, yielding different domain structures from vertebrate HS [58], [59]. This diversity most likely results in the duality of both non-specific ionic interactions occurring between HS and positively charged ligands *versus* highly specific interactions of particular ligands with certain HS species [60]–[63]. Specificity for particular HS structures has been documented for several virus-HS interactions [38], [64]. Therefore, it was possible that mutations affecting the charge balance of E2 selected on different cell types might exhibit different types of HS interaction. While the particular contribution of HS binding to alphavirus infection is not fully characterized beyond increases in virus association with cells [17], [21], [24], it is possible that structural differences in HS chains, their attachment to core proteins or their associations with other cell surface factors may be involved in the selective advantage provided by a particular virus mutation *in vitro*.

Comparing CHO and C6/36 cell passage series, we obtained one common mutation altering charge (E2-79 E-K), three CHO-only mutations (E255 G-R, E2-159 S-R and E2-168 E-K) and one C6/36-only mutation (deletion of E2-166E). Neither the V to A substitution at E2-264, nor the H to Y substitution at E2-99, would be anticipated to have considerable impact upon electrostatic potential; therefore, these were not considered. Interestingly, the E2-159R selected in these studies was previously selected during passage of CHIKV on Vero primate kidney fibroblast cells [65], the E2-166K was selected on HeLa human adenocarcinoma cells [36], and the E2-82R in the vaccine strain was selected on MRC-5 human fetal fibroblast cells [9], although none of these studies deliberately selected for increased infectivity or HS interaction. Therefore, common mutations can be selected between evolutionarily divergent hosts (E2-79 E-K in hamster and mosquito, and E2-159 S-R in monkey and mouse) while it is possible that others are unique to particular cells or species. While not considered in our studies, it is possible that the number of different mutations selected would be increased by using cells from different tissue types reflecting the diversity of tissue specific HS structure described above.

To determine whether or not the mutations identified were unique to cell culture-adapted virus populations, we examined these positions in an alignment of 200 pE2 sequences available in GenBank and believed to be from isolates minimally or unpassaged before sequencing (Gardner *et al.*, unpublished observations). Only two of the mutations selected in our study were present in any of these isolates: four isolates had E2-55R (ADV31296, AEE60792, AEE60797 and BAH97931) and 10 isolates had the E2-264A (AEX25348, AEX25344, AEX25346, AEE60795, AEE60793, AEE60796, AEE60794, AEE60792, CCA61129 and CCA61128). Whether or not these residues are present in naturally circulating viruses or represent cell culture adaptive mutations in the minimally passaged strains remains to be determined.

With the exception of LR-Δ166E, all of the E2 mutants were attenuated for the ability to cause MSD in mice. Although all of the viruses were clearly able to replicate at the site of inoculation in the footpad, in general their replication in MST was reduced. The virus mutants fell into three groups of disease severity with LR-Δ166E disease indistinguishable from CHIKV-LR; LR-168K, LR-55R and LR-159R eliciting a biphasic MSD similar to CHIKV-LR but with reduced disease; and LR-166K, LR-82R and LR-79K eliciting either a minor monophasic MSD (LR-166K) or no detectable MSD (LR-82R and LR-79K). As suggested by studies with other alphaviruses [17], [20], [48], the dependence upon HS for infectivity prevents efficient spread of CHIKV *in vivo*. In the current studies, this led to decreased levels of inflammatory chemokines such as MCP-1, MIG and IP-10 that are associated with CHIKV-induced disease. Further, the most attenuated viruses elicited the lowest levels of these factors. It was not surprising that the levels of IP-10 and MCP-1 correlated with attenuation, as these cytokines are chemoattractants for monocytes/macrophages which have been shown to be important for disease mice [39], [41] and humans, especially during the acute phase of disease [42]–[44], [66], and can be associated with higher CHIKV loads [44]. MIG and IL-12 are involved in T cell recruitment and differentiation respectively and have been shown in some human cohorts to be elevated during infection [42]–[45], [66], and CD4+ T cells have been shown to contribute to hind limb swelling in mice [67]. IL-1α, IFN-γ and IL-2 were increased in the serum of mutant viruses similarly to wild-type CHIKV-LR and several of these cytokines were also shown to be upregulated during human infections [41], [43]–[45], [66]. It remains to be determined if elevation of these cytokines is associated with the attenuated phenotype and/or the induction of the protective immune response. Taken together, the cytokine profiles in mice correlated with virulence and for many of the cytokines are similar to profiles in humans following CHIKV-LR infection.

We attempted to associate *in vitro* measurements of HS infection dependence with virulence, examining virus specific infectivity, infection efficiency in the absence of HS or all GAGs, plaque size, resistance of infectivity to competition with increasing salt concentrations, sensitivity to competition with heparin, and infection sensitivity to digestion of cell surfaces with heparinases. Each of these assays has previously been used to compare HS infectivity dependence of alphaviruses (e.g., [17], [19], [21], [22], [48], [68]). All viruses attenuated in comparison with CHIKV-LR did exhibit increased specific infectivity on at least one of the four cell types tested and this was associated with similar infectivity diminution in cells genetically deficient in GAG synthesis. LR-Δ166E dependence on HS was significantly less than the other mutants but much greater than CHIKV-LR indicating that at least partial dependence upon HS for infectivity is not invariably associated with attenuation. Interestingly, in contrast with the SINV-K70 mutant whose infectivity appeared to depend solely upon HS among GAGs, all CHIKV mutants exhibited a minor dependence of infectivity upon GAGs other than HS evidenced by slightly increased infectivity on HS-deficient pgsD-677 cells *versus* GAG-deficient pgsA745 cells. This suggests the possibility of differences in attachment/entry mechanisms of SINV *versus* CHIKV.

Disruption of infection with salt did not distinguish between attenuated mutants with each exhibiting moderate decrease at 200 mM and ∼90% decrease in infectivity at 250 mM and above. Interestingly, LR-Δ166E, although sensitive to genetic deficiency in HS and all GAGs, was insensitive to all salt concentrations used, similar to CHIKV-LR. Furthermore, digestion of HS chains with Hep I, II or III did not distinguish the three groups of attenuation phenotypes. Likely reflecting the different substrate specificities of the three enzymes, differential sensitivity to the three heparinases can distinguish between virulence phenotypes with other alphaviruses with genetic differences in HS binding domains [17], [29]. In these studies, CHIKV-LR was insensitive to all three heparinases, while LR-166K, LR-168K, LR-55R, LR-159R and LR-82R were partially sensitive to Hep I, highly sensitive to Hep II and partially sensitive to Hep III. Notably, LR-79K was significantly more sensitive to Hep I and Hep III than the other E2 mutants. LR-Δ166E, again, exhibited an intermediate dependence phenotype with all three heparinases. SINV-K70, in comparison, was highly sensitive to all three heparinases, again suggesting differences in GAG interactions between SINV and CHIKV mutants. In contrast with these assays, blocking of infectivity by reaction of virus particles with soluble heparin clearly distinguished LR-166K, LR-82R and LR-79K from the other viruses. Small plaque size and lack of change in size with addition of heparin to the overlay provided a similar distinction.

Comparison of location in an electrostatic map of the CHIKV heterotrimeric spike complex revealed that mutations that were more attenuating to disease in mice appeared to create additional positive charge that was more exposed to the exterior of the spike (e.g., E2-79K, E2-82R and E2-166K as opposed to E2-55R, E2-Δ166E or E2-159R). The lack of any attenuating phenotype with E2- E2-Δ166E was surprising, especially since the electrostatic model of this mutant suggested that the deletion should lead to increased electrostatic potential of E2 similar to that of the other E2 amino acid substitution mutants listed in Table 1 which demonstrated various degrees of attenuation. Clearly, the interpretation of the electrostatic potential maps of particular E2 mutant proteins will depend upon confirmation with *in vitro* assessment of GAG dependency.

Our data for the E2-166K mutation, which we tested because of its identical location to the selected E2-Δ166E mutation, indicate that it confers efficient attachment to HS. This mutation was selected by passage in a context where the authors concluded that the mutation increased virus resistance to the OAS3 antiviral protein, possibly by conferring a rapid entry phenotype [36]. Many of the HS-binding E2 mutants for SINV and VEEV were originally selected and identified as rapid entry mutants and only later shown to confer efficient HS binding [17], [24], [69]–[72]. The relationship of HS binding, rapid entry and antiviral resistance is unclear. However, if the LR-166K virus effectively antagonized the antiviral response as suggested by Henrik Gad *et al.* [36], one would expect the virus to be more virulent *in vivo* instead of more attenuated compared to CHIKV-LR and thus this phenotype may be an *in vitro* artifact or a localized phenomenon.

Overall, our data suggest that positively charged amino acid substitutions in CHIKV E2 that result in small plaques and efficient competition with heparin will be highly attenuated in the adult mouse model of MSD. The LR-166K, LR-82R and LR-79K viruses were very similar in terms of the primary *in vitro* correlates of attenuation: plaque size and heparin sensitivity. In addition, LR-79K was dramatically more attenuated for MSD than LR-82R in the severely immunocompromised STAT1-deficient mice. Testing in this model will refine choices between mutants with similar *in vitro* characteristics and sensitivity to all three heparinases may indicate the highest degree of *in vivo* attenuation within this group. Furthermore, combinations of such mutations can be tested to improve immunogenicity and/or stabilize attenuation. One question raised by these studies is whether or not this paradigm for deliberate mutation selection will be applicable to other alphaviruses and/or other arboviruses. We have detected differences between SINV and CHIKV mutants in dependency upon HS *versus* other GAGs for infectivity and it is unclear how this phenotype correlates with attenuation *in vivo* especially when evaluating the results of assays that are not specific to a particular GAG. It is likely that the specific combination of *in vitro* assays most correlated with attenuation must be determined for each virus type.

In summation, these studies demonstrate that very limited passage of CHIKV is sufficient to generate HS binding mutations that are highly attenuating but retain immunogenicity, and would make satisfactory candidates for testing in a LAV. Furthermore, using this approach, we have identified E2-79K as a single site mutation that is highly attenuating for MSD and stable even in a severely immunocompromised model of disease. It is possible that attenuating mutations present in other areas of vaccine genomes (e.g., nonstructural or non-translated region mutations in the YFV 17D vaccine or the VEEV TC83 vaccine [73]) would require further passage to accrue. Therefore, rational LAV creation may benefit from multiple selection strategies with an initial screen identifying mutants meeting the criteria we have outlined for HS binding-mediated attenuation followed by additional passage on the same cell type or others or introduction of mutations identified with other alphaviruses that could be reasonably transferred to CHIKV.

## Supporting Information

Figure S1 **HS-dependent infectivity of CHIKV E2 mutants.** CHIKV E2 mutants were evaluated for dependency on HS for infectivity by infectivity on cells deficient in either GAGs or HS. (A) CHOK1, pgsA745 (GAG negative) and pgsD677 (HS negative) cells were infected with virus and at 24 h p.i. fixed with 4% PFA and stained for CHIKV antigen. Percent infection on pgsA745 and pgsD677 cells was normalized to infectivity on CHOK1 cells that was set to 100%. Error bars represent standard deviation. *p<0.001 compared to CHOK1 cells.
(TIF)

Figure S2 **Attenuation of CHIKV E2 mutants in immunocompromised mouse model.** The most attenuated CHIKV-E2 mutants in 21d CD1 mice were further tested for attenuation in an immunocompromised mouse model. STAT129 mice (n = 4–5) were infected subcutaneously in the rear footpad with either 10^5^ genome equivalents of either (A) CHIKV-LR, (B) LR-82R or (C) LR-79K. The metatarsal region (height and width) was measured daily and fold change was calculated based on pre-infection footpad area. Individual mice are graphed with the intensity of shading indicates the proportion of mice with overlapping levels of hind-limb swelling.
(TIF)

Table S1 **Serum cytokine levels at two days post-infection.** *p<0.05*; **p<0.01; ***p<0.001; measured in pg/ml.
(DOCX)
