# Supplementary figures and images for: Deliberate Attenuation of Chikungunya Virus by Adaptation to Heparan Sulfate-Dependent Infectivity: A Model for Rational Arboviral Vaccine Design
Source: PLoS Negl Trop Dis. 2014 Feb 20;8(2):e2719. doi: 10.1371/journal.pntd.0002719 (PMC3930508; doi:10.1371/journal.pntd.0002719)

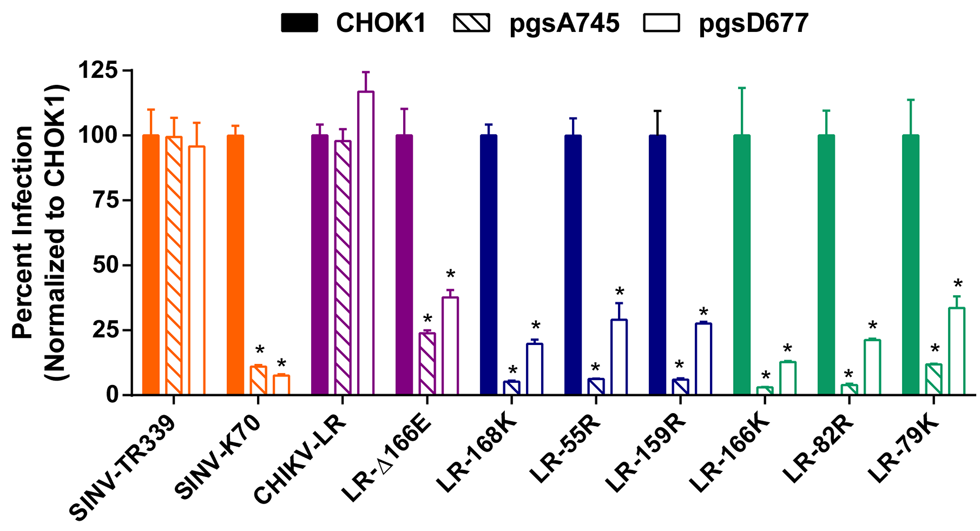

Supplement: Figure S1 — HS-dependent infectivity of CHIKV E2 mutants. CHIKV E2 mutants were evaluated for dependency on HS for infectivity by infectivity on cells deficient in either GAGs or HS. (A) CHOK1, pgsA745 (GAG negative) and pgsD677 (HS negative) cells were infected with virus and at 24 h p.i. fixed with 4% PFA and stained for CHIKV antigen. Percent infection on pgsA745 and pgsD677 cells was normalized to infectivity on CHOK1 cells that was set to 100%. Error bars represent standard deviation. *p<0.001 compared to CHOK1 cells. (TIF) [file pntd.0002719.s001.tif]

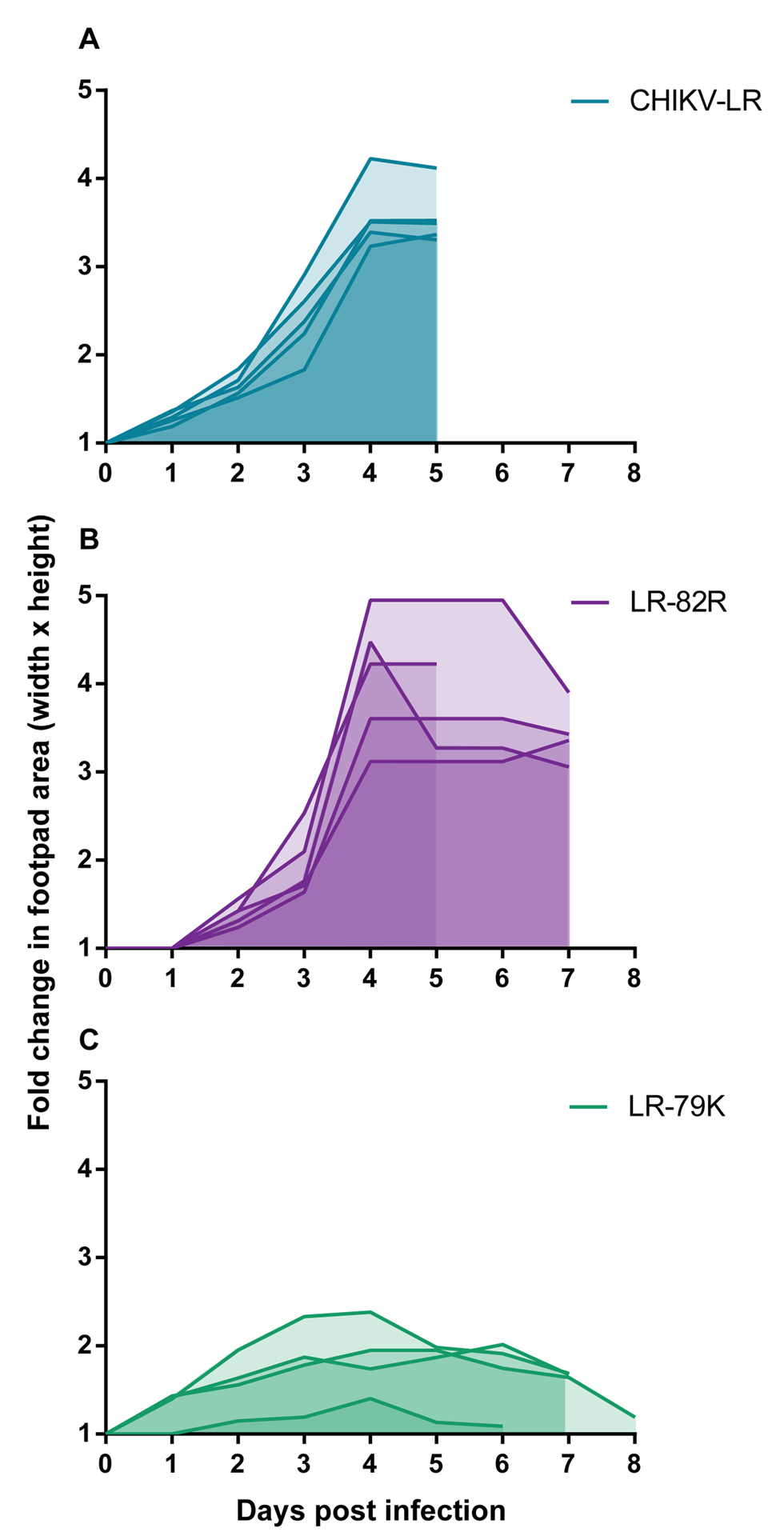

Supplement: Figure S2 — Attenuation of CHIKV E2 mutants in immunocompromised mouse model. The most attenuated CHIKV-E2 mutants in 21d CD1 mice were further tested for attenuation in an immunocompromised mouse model. STAT129 mice (n = 4–5) were infected subcutaneously in the rear footpad with either 105 genome equivalents of either (A) CHIKV-LR, (B) LR-82R or (C) LR-79K. The metatarsal region (height and width) was measured daily and fold change was calculated based on pre-infection footpad area. Individual mice are graphed with the intensity of shading indicates the proportion of mice with overlapping levels of hind-limb swelling. (TIF) [file pntd.0002719.s002.tif]
