# Supplementary material for: Deliberate Attenuation of Chikungunya Virus by Adaptation to Heparan Sulfate-Dependent Infectivity: A Model for Rational Arboviral Vaccine Design
Source: PLoS Negl Trop Dis. 2014 Feb 20;8(2):e2719. doi: 10.1371/journal.pntd.0002719 (PMC3930508; doi:10.1371/journal.pntd.0002719)
Supplement: Table S1 — Serum cytokine levels at two days post-infection. p<0.05; **p<0.01; ***p<0.001; measured in pg/ml. (DOCX) [file pntd.0002719.s003.docx]

| **Supplementary Table 1:** Serum cytokine levels (pg/ml) at 48 h p.i. | | | | | | | | | |
| --- | --- | --- | --- | --- | --- | --- | --- | --- | --- |
|  | **Mock** | **LR-WT** | **LR-d166** | **LR-168K** | **LR-55R** | **LR-159R** | **LR-166K** | **LR-82R** | **LR-79K** |
| FGF basic | 64 ± 20 | 68 ± 23 | 81 ± 17 | 63 ± 4 | 85 ± 26 | 78 ± 19 | 85 ± 17 | 67 ± 10 | 97 ± 40 |
| GM-CSF | 12 ± 0 | 13 ± 1 | 12 ± 2 | 12 ± 1 | 13 ± 2 | 13 ± 3 | 16 ± 2 | 13 ± 1 | 13 ± 3 |
| **IFN-γ** | 25 ± 7 | **52 ± 18*** | **47 ± 12*** | **38 ± 2*** | **41 ± 10*** | **48 ± 12*** | **41 ± 10*** | **40 ± 3**** | **44 ± 12*** |
| IL-1β | 15 ± 1 | 24 ± 2 | 26 ± 1 | 16 ± 2 | 19 ± 5 | 21 ± 3 | 20 ± 2 | 17 ± 1 | 19 ± 2 |
| **IL-2** | 19 ± 2 | **62 ± 14**** | **38 ± 4*** | 20 ± 1 | **44 ± 4***** | **34 ± 10*** | 32 ± 13 | 23 ± 2 | 25 ± 4 |
| IL-4 | 35 ± 3 | 41 ± 11 | 37 ± 5 | 41 ± 5 | 42 ± 9 | 42 ± 15 | 38 ± 17 | 38 ± 7 | 35 ± 11 |
| IL-6 | 11 ± 0 | 17 ± 2 | 21 ± 3 | 13 ± 1 | 15 ± 4 | 18 ± 1 | 14 ± 0 | 13 ± 2 | 16 ± 4 |
| IL-10 | 266 ± 60 | 314 ± 17 | 338 ± 14 | 287 ± 20 | 330 ± 36 | 339 ± 12 | 390 ± 91 | 280 ± 67 | 326 ± 21 |
| IL-13 | 46 ± 8 | 50 ± 5 | 41 ± 2 | 40 ± 3 | 47 ± 3 | 48 ± 10 | 43 ± 2 | 42 ± 1 | 50 ± 11 |
| IL-17 | 13 ± 4 | 22 ± 2 | 16 ± 1 | 15 ± 3 | 23 ± 1 | 20 ± 3 | 23 ± 5 | 20 ± 4 | 21 ± 7 |
| KC | 184 ± 61 | 188 ± 26 | 170 ± 12 | 155 ± 10 | 175 ± 18 | 196 ± 52 | 189 ± 43 | 147 ± 10 | 195 ± 47 |
| MIP-1α | 8 ± 2 | 12 ± 2 | 23 ± 14 | 9 ± 1 | 13 ± 4 | 21 ± 7 | 15 ± 5 | 15 ± 4 | 22 ± 13 |
| TNF-α | 15 ± 1 | 17 ± 1 | 16 ± 1 | 12 ± 1 | 16 ± 1 | 15 ± 1 | 14 ± 3 | 14 ± 1 | 14 ± 0 |
| VEGF | 67 ± 6 | 58 ± 14 | 61 ± 3 | 48 ± 3 | 62 ± 3 | 63 ± 7 | 79 ± 12 | 56 ± 8 | 62 ± 10 |
| * p<0.05; **p<0.01; ***p<0.001 | | | | | | | | | |
